# Differences in the Composition of the Human Antibody Repertoire by B Cell Subsets in the Blood

**DOI:** 10.3389/fimmu.2014.00096

**Published:** 2014-03-19

**Authors:** Eva Szymanska Mroczek, Gregory C. Ippolito, Tobias Rogosch, Kam Hon Hoi, Tracy A. Hwangpo, Marsha G. Brand, Yingxin Zhuang, Cun Ren Liu, David A. Schneider, Michael Zemlin, Elizabeth E. Brown, George Georgiou, Harry W. Schroeder

**Affiliations:** ^1^Department of Microbiology, University of Alabama at Birmingham, Birmingham, AL, USA; ^2^Department of Molecular Biosciences, University of Texas at Austin, Austin, TX, USA; ^3^Laboratory for Neonatology and Pediatric Immunology, Department of Pediatrics, Philipps-University, Marburg, Germany; ^4^Department of Chemical Engineering, University of Texas at Austin, Austin, TX, USA; ^5^Department of Biomedical Engineering, University of Texas at Austin, Austin, TX, USA; ^6^Department of Medicine, University of Alabama at Birmingham, Birmingham, AL, USA; ^7^Department of Biochemistry and Molecular Genetics, University of Alabama at Birmingham, Birmingham, AL, USA; ^8^Department of Epidemiology, University of Alabama at Birmingham, Birmingham, AL, USA

**Keywords:** human antibody repertoire, CDR-H3, B cells subsets

## Abstract

The vast initial diversity of the antibody repertoire is generated centrally by means of a complex series of V(D)J gene rearrangement events, variation in the site of gene segment joining, and TdT catalyzed N-region addition. Although the diversity is great, close inspection has revealed distinct and unique characteristics in the antibody repertoires expressed by different B cell developmental subsets. In order to illustrate our approach to repertoire analysis, we present an in-depth comparison of V(D)J gene usage, hydrophobicity, length, D_H_ reading frame, and amino acid usage between heavy chain repertoires expressed by immature, transitional, mature, memory IgD^+^, memory IgD^−^, and plasmacytes isolated from the blood of a single individual. Our results support the view that in both human and mouse, the H chain repertoires expressed by individual, developmental B cell subsets appear to differ in sequence content. Sequencing of unsorted B cells from the blood is thus likely to yield an incomplete or compressed view of what is actually happening in the immune response of the individual. Our findings support the view that studies designed to correlate repertoire expression with diseases of immune function will likely require deep sequencing of B cells sorted by subset.

## Introduction

Production of a highly diverse, polyclonal immunoglobulin repertoire plays a central role in the ability of B cells to produce antibodies specific to a diverse range of foreign and self-antigens (1, 2). The antigen-binding sites of these antibodies are created by the juxtaposition of six hypervariable loops, termed complementarity determining regions (CDRs): three from the heavy (H) and three from the light (L) chain V domains. Because the third CDR of the H chain, termed CDR-H3 (2–5), is the direct product of V(D)J joining and N-region addition, it is the most variable component of the pre-immune immunoglobulin repertoire. The location of CDR-H3 at the center of the antigen-binding site allows this interval to play a key role in antigen recognition and binding (6–8).

Developing B cells pass through a series of checkpoints designed to test the functionality and antigen specificity of the immunoglobulin (9–14). In adults, this process begins in the bone marrow, and then continues in the periphery where it is heavily influenced by exposure to both self and foreign antigens. Immature B cells are released into the blood and in the periphery pass through a transitional stage prior to entering specific anatomic sites, such as the splenic marginal zone and the splenic and lymph node follicles (15, 16). Maturation is associated with the co-expression of IgM and IgD (17). Mature cells exposed to antigen can become either memory cells or plasmacytes. Both types of cells circulate through the blood on their way to their specific anatomic niches (18–21). IgM bearing memory cells can be divided into two populations, those that express IgD concurrently and those that do not (22–25). The IgM^+^IgD^−^ memory B cell population includes conventional, follicular B cells, whereas the IgM^+^IgD^+^memory B cell population includes marginal zone-like B cells that play a more immediate role in response to foreign antigens (26–28).

Recent studies in mice have shown that the composition of CDR-H3 exhibits preferred patterns in amino acid composition, length, and charge distribution that differ by developmental stage and B cell subset (29–33). These categorical constraints are initially imposed by natural selection of the germline V, D, and J gene sequence; and alteration of the sequence of these gene segments can give rise to dramatically different CDR-H3 repertoires (34–36). D gene sequence-specific changes in CDR-H3 content lead to altered patterns of B cell development, antigen-specific antibody production, and levels of protection against infectious agents (31, 37, 38), which underscores the important role played by the composition of the CDR-H3 repertoire in the regulation and function of the humoral immune response.

Given the importance of CDR-H3 to antigen recognition and antibody specificity, and the observation that CDR-H3 content can differ by peripheral developmental stage in the mouse; we sought to test whether V(D)J usage and CDR-H3 content would also differ by developmental stage in human. We used surface expression of CD19, CD27, IgD, CD24, and CD38 expression to identify and sort immature, transitional, mature, memory IgD^+^, memory IgD^−^ B cell subsets, and plasmacytes from the blood of a healthy female subject. We then used RT-PCR followed by Roche GS-FLX 454 deep sequencing to clone and sequence Cμ and Cγ-containing transcripts from the sorted cells. As in the mouse, we found that the distribution of V, D, and J utilization, and CDR-H3 length, amino acid usage, and average hydrophobicity differed between developmentally and functionally distinct B cell subsets. We conclude that studies of differences between healthy individuals and patients with diseases referable to the humoral immune response will likely require comparisons of the B cell repertoire by subset.

## Materials and Methods

### Subject description and isolation of B cell subsets

One healthy female subject, age 56, was recruited for antibody repertoire high throughput sequencing using the 454 platform. The subject is Caucasian, a lifelong native of the state of Alabama, and was without a history of illness or repeated infection that could be related to abnormal immune function. The complete blood count was well within normal limits. Serum immunoglobulin levels were IgM 382, IgG 1,680, and IgA 368 mg/dL, respectively. Venous blood (100 cm^3^) was drawn by routine venipuncture and mononuclear cells were isolated using Ficoll-Paque Plus (GE Healthcare). CD19^+^ magnetic beads (Miltenyi Biotec MACS) were used to enrich for B cells. These CD19^+^ cells were further fractionated by CD27^±^ populations using CD27 magnetic beads (Miltenyi Biotec MACS) according to the manufacturer’s protocol. CD19^+^CD27^+^ B cells were stained with CD19 APC_780_ (eBioscience), CD27 PE–Cy7 (BD Pharmingen), CD24 APC (BioLegend), and IgD FITC (Southern Biotech), and sorted into IgD^+^ memory B cells (CD19^+^/CD27^+^/IgD^+^/CD24^+^), IgD^−^ memory B cells (CD19^+^/CD27^+^/IgD^−^/CD24^+^), and plasmacytes (CD19^+^/CD27^+^/CD24^−^) using a high speed sorting cytometer (FACSAria III; Becton Dickinson). CD19^+^/CD27^−^ B cells were stained with CD19 APC_780_ (eBioscience), CD24 APC (BioLegend), CD38 PE (BioLegend), and IgD FITC (Southern Biotech) and sorted into mature/naïve (CD19^+^/CD27^−^/IgD^+^/CD38^+^/CD24^+^), transitional (CD19^+^/CD27^−^/IgD^+^/CD38^+++^/CD24^+++^), and immature (CD19^+^/CD27^−^/IgD^−^) B cell subsets. Each B cell subset was then individually resuspended in 1 mL TRI reagent (Ambion) and archived at −80°C until processed for total RNA extraction. This work was performed in accordance with an Institutional Review Board approved protocol and informed consent was obtained from the subject at the University of Alabama at Birmingham, Birmingham, AL, USA.

### Generation of IgH libraries

For RNA extraction, 0.2 mL chloroform was added to the 1 mL sample, vortexed for 15 s, left to stand at room temperature for 5 min, then spun at 12,000 × *g* for 10 min at 4°C. The aqueous phase (~400 μL) was removed and to this an equal volume of 70% ethanol was added and then mixed by pipetting. This was applied immediately to an RNA-binding silica spin-column and subsequently processed according to the manufacturer’s protocol (Qiagen RNeasy micro column; catalog no. 74004). Purified total RNA was eluted in 14 μL RNase-free water. Oligo-dT primer was used to generate first-strand cDNA from ~100 ng input RNA using the SuperScript RT II synthesis kit (Invitrogen; catalog no. 11904-018) per the manufacturer’s protocol.

FastStart high fidelity PCR system (Roche; catalog no. 03-553-361-001) and an equimolar mix of eight optimized VH-FWD primers previously described for human IgH amplification (39, 40) coupled with a multiplex of 10-nucleotide uniquely barcoded CH-REV primers: IgM-rev, 5′-10 nt ID-GGTTGGGGCGGATGCACTCC-3′, and IgG-all-rev, 5′-10 nt ID-SGATGGGCCCTTGGTGGARGC-3′ were used to amplify V(D)JCμ and V(D)JCγ cDNAs from the cDNA template. Cycling conditions were as follows: 95°C denaturation for 3 min; 92°C for 1 min, 50°C for 1 min, 72°C for 1 min for 4 cycles; 92°C for 1 min, 55°C for 1 min, 72°C for 1 min for 4 cycles; 92°C for 1 min, 63°C for 1 min, 72°C for 1 min for 22 cycles; 72°C for 7 min. PCR amplicons were gel-purified (Zymo Research) before sequencing.

### High-throughput sequencing of IgH repertoires and bioinformatic analysis

The University of Texas Genomics Sequencing and Analysis Facility performed Roche GS-FLX 454 deep sequencing. CH-REV barcodes were examined to verify the integrity of each library after filtering raw data for read quality. Sequences were submitted to the ImMunoGeneTics (IMGT) database and IMGT/high V-QUEST web-based analysis tool (version 1.0.3) (41). The 11 CSV text files outputted by IMGT/highV-QUEST were then imported into IgAT immunoglobulin analysis tool for further deconstruction (42). Differences between populations were assessed, where appropriate, by Student’s *t*-test, two tailed; Fisher’s exact test, two tailed and *d*; χ^2^, or Levene’s test for the homogeneity of variance. Analysis was performed with PRISM version 5 (Graph Pad). The standard deviation accompanies mean. Raw 454 sequence files were deposited to the NCBI Sequence Read Archive (Accession SRP037774).

## Results

### Isolation of B lineage cells and 454 high-throughput sequencing of IgH transcripts from peripheral blood

**Figure 1:**
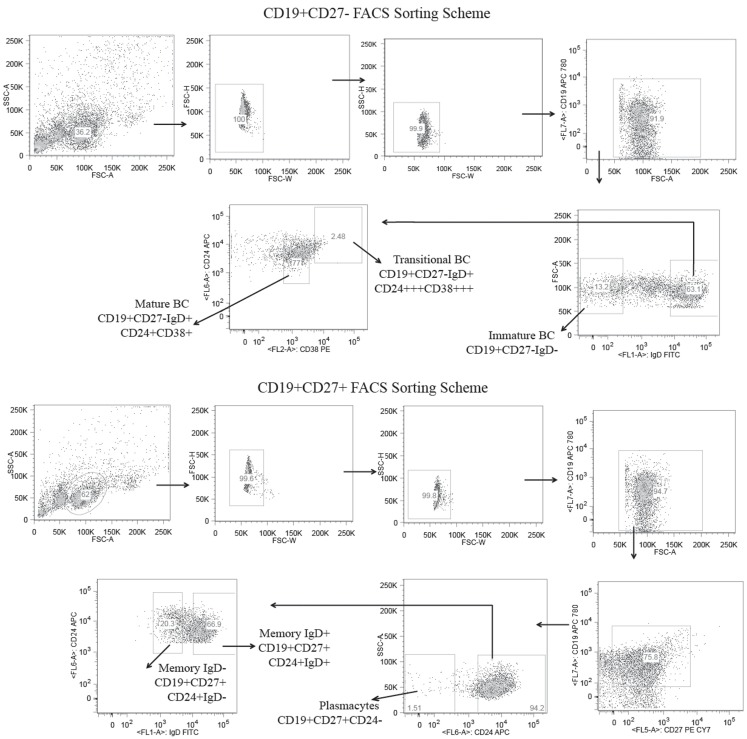
**Flow cytometric gates for the collection of six distinct B cell lineage populations from the peripheral blood of a healthy adult human subject**. B cells were separated from the total lymphocytes using CD19^+^ magnetic beads and further separated into CD27^±^ populations using CD27 magnetic beads. The CD19^+^CD27^+^ B cells were stained with CD19 APC_780_, CD27 PE-Cy7, CD24 APC, and IgD FITC and sorted into IgD+ memory B cells (CD19^+^/CD27^+^/IgD^+^/CD24^+^), IgD^−^ memory B cells (CD19^+^/CD27^+^/IgD^−^/CD24^+^), and plasmacytes (CD19^+^/CD27^+^/CD24^−^) using the high speed sorting cytometer. The CD19^+^/CD27^−^ B cells were stained with CD19, APC 780, CD24 APC, CD38 PE, and IgD FITC, and sorted into mature (CD19^+^/CD27^−^/IgD^+^/CD38^+^/CD24^+^), transitional (CD19^+^/CD27^−^/IgD^+^/CD38^+++^/CD24^+++^), and immature (CD19^+^/CD27^−^/IgD^−^) B cell subsets.

CD19^+^ cells bearing the cell surface markers characteristic of immature, transitional, mature, memory IgD^+^, memory IgD^−^, and plasmacytes were isolated from the blood of a healthy female subject (43–47) (Figure 1). Following total RNA extraction, PCR was used to amplify cDNA copies of V(D)JCμ and V(D)JCγ transcripts using optimized VH-FWD primers previously described for human IgH amplification (39, 40). We obtained a total of 15,433 immature, 37,396 transitional, 47,781 mature, 43,558 memory IgD^+^, 28,142 memory IgD^−^, and 43,824 plasmacyte unique and in-frame IgH heavy chain reads. Of these, we obtained 1,240 immature, 1,354 transitional, 1,250 mature, 1,244 memory IgD^+^, 833 memory IgD^−^, and 1,714 plasmacyte reads that were of sufficient length to be identified as Igμ sequences, and 1,879 memory IgD^−^ and 3,347 plasmacyte reads that were of sufficient length to be identified as Igγ sequences. All of the unique Igμ and Igγ reads were deconstructed to assess the presence and extent of changes in these repertoires that had occurred as B cells progressed through the various developmental checkpoints.

### The immature B cell receptor repertoire utilizes shortest contribution of germline gene VJ segments and favors V1–18, D2–15, D4–23, and D5–12

**Figure 2:**
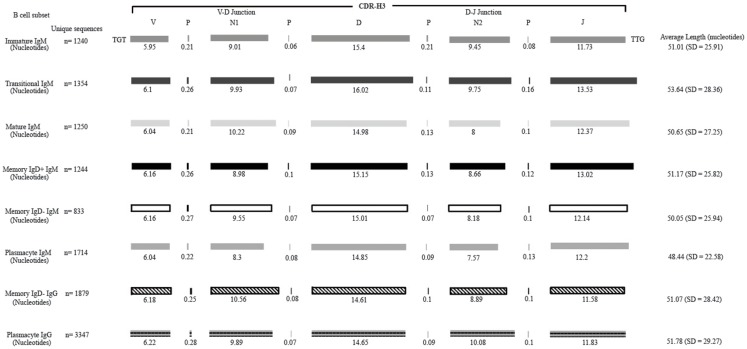
**Deconstruction of the contributing components to CDR-H3 length in Igμ and Igγ reads containing identifiable D_H_ gene segments as a function of B cell development in the peripheral blood**. The contributions of nucleotides provided by the V_H_, D_H_, and J_H_ gene segments, by P junctions, and by the extent of N addition at the V_H_ → D_H_ and D_H_ → J_H_ junctions to the CDR-H3 length are illustrated. The IgAT (42) identified the CDR-H3 as amino acids 105–117, according to the IMGT unique numbering system. The average length was calculated with the components of the CDR-H3, namely the V length, P-nucleotides 3′ of the V, N1 nucleotides, P-nucleotides 5′ of D, D length, P-nucleotides 3′ of D, N2 nucleotides, P-nucleotides 3′ of J, and J length. The deconstructed CDR-H3 segments shown are of CDR-H3 sequences with identifiable D_H_ gene segments. The reported average length is the average length of all CDR-H3 sequences (with the identifiable D_H_ and without identifiable D_H_ gene segments) accompanied by the standard deviation.

**Figure 3:**
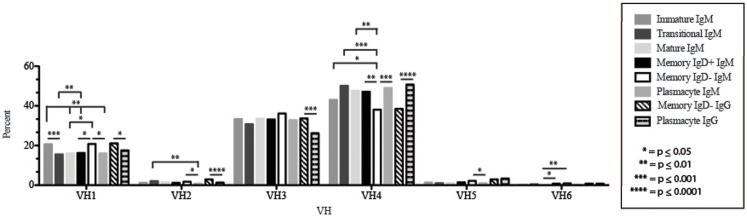
**V_H_ gene segment usage in Igμ and Igγ transcripts from selected B cell populations in the blood of a normal, healthy human**. V_H_ gene segments are arranged according to their position relative to the J_H_ locus in the genome. Percent of unique, in-frame sequences using the V_H_ gene segment specified in the peripheral blood from immature, transitional, mature, memory IgD^+^, memory IgD^−^ B cells, and plasmacytes are displayed. All comparisons were made using χ^2^-test or Fisher’s exact test as appropriate. Significant differences among each fraction in the different mice are indicated by asterisks: *p ≤ 0.05, **p ≤ 0.01, ***p ≤ 0.001, ****p ≤ 0.0001.

**Figure 4:**
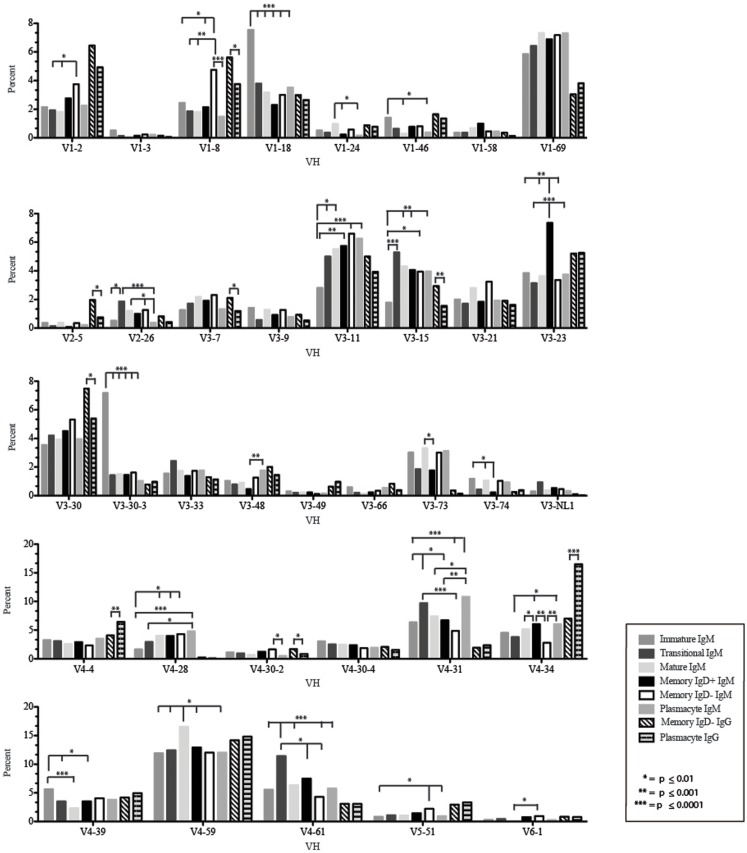
**Individual V_H_ gene segment usage in Igμ and Igγ transcripts from selected B cell populations in the blood of a normal, healthy human**. Percent of unique, in-frame sequences using the individual V_H_ gene segments specified in the peripheral blood from immature, transitional, mature, memory IgD^+^, memory IgD^−^ B cells, and plasmacytes are displayed. All comparisons were made using χ^2^-test or Fisher’s exact test as appropriate. Significant differences among each fraction in the different mice are indicated by asterisks: *p ≤ 0.01, **p ≤ 0.001, ***p ≤ 0.0001.

**Figure 5:**
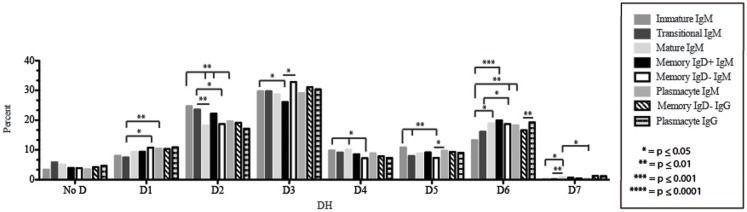
**D_H_ family usage in Igμ and Igγ transcripts from selected B cell populations in the blood of a normal, healthy human**. The percent of sequences using members of the specified D_H_ family among in-frame reads obtained from the peripheral blood from immature, transitional, mature, memory IgD^+^, memory IgD^−^ B cells, and plasmacytes are displayed. All comparisons were made using χ^2^-test or Fisher’s exact test as appropriate. Significant differences among each fraction in the different mice are indicated by asterisks: *p ≤ 0.05, **p ≤ 0.01, ***p ≤ 0.001, ****p ≤ 0.0001.

**Figure 6:**
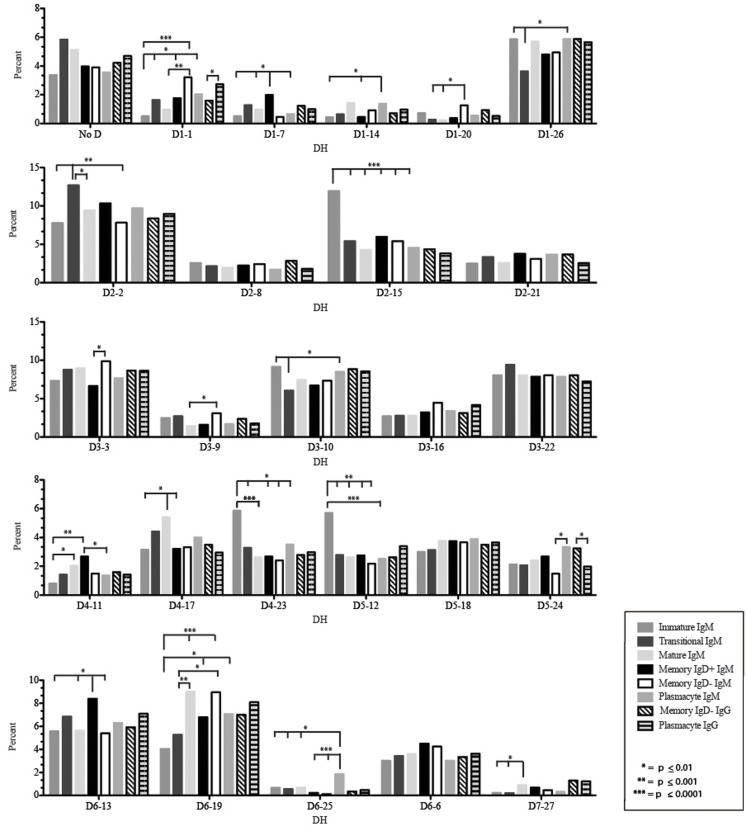
**Individual D_H_ gene segment usage in Igμ and Igγ transcripts from selected B cell populations in the blood of a normal, healthy human**. Percent of unique, in-frame reads using the individual D_H_ gene segments specified in the peripheral blood from immature, transitional, mature, memory IgD^+^, memory IgD^−^ B cells, and plasmacytes are displayed. All comparisons were made using χ^2^-test or Fisher’s exact test as appropriate. Significant differences among each fraction in the different mice are indicated by asterisks: *p ≤ 0.01, **p ≤ 0.001, ***p ≤ 0.0001.

**Figure 7:**
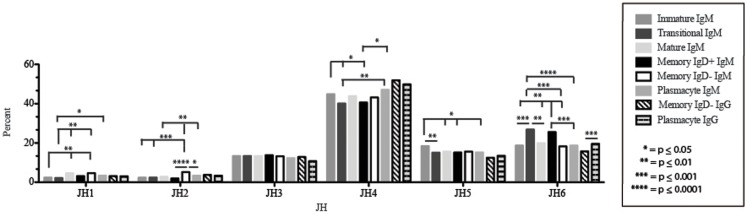
**J_H_ usage in Igμ and Igγ transcripts from selected B cell populations in the blood of a normal, healthy human**. The percent of sequences using J_H_1 through J_H_6 among in-frame reads cloned from the peripheral blood from immature, transitional, mature, memory IgD^+^, memory IgD^−^ B cells, and plasmacytes are displayed. All comparisons were made using χ^2^-test or Fisher’s exact test as appropriate. Significant differences among each fraction in the different mice are indicated by asterisks: *p ≤ 0.05, **p ≤ 0.01, ***p ≤ 0.001, ****p ≤ 0.0001.

The immature B cell subset is primarily composed of recent bone marrow emigrants. It expressed a highly diverse repertoire that differed from the subsequent transitional stage in that it contained the smallest contribution of germline V and J gene sequence to the CDR-H3 region (Figure 2). By family, V_H_4 gene segments contributed the most, followed by V_H_3, V_H_1, V_H_5, V_H_2, and V_H_6 (Figure 3). By individual V gene segments, V1–18, V1–69, V3–73, and V4–59 were most common. Across subsets, the immature B cell subset was enriched for V1–18, V3–30–3, and V3–74 (Figure 4). By D_H_ family, D_H_3 was the most common, followed by D_H_2 and D_H_6 (Figure 5). By individual D gene segment, D2–2, D3–3, D3–22, D6–13, and D6–19 were favored. Across subsets, D1–26, D2–15, D3–10, D4–23, and D5–12 were more commonly used in the immature B cell lineage (Figure 6). By J_H_ gene segment, J_H_4 was the most common, followed by J_H_6, J_H_5, and J_H_3. Across subsets, immature B cells used J_H_5 more frequently (Figure 7).

**Figure 8:**
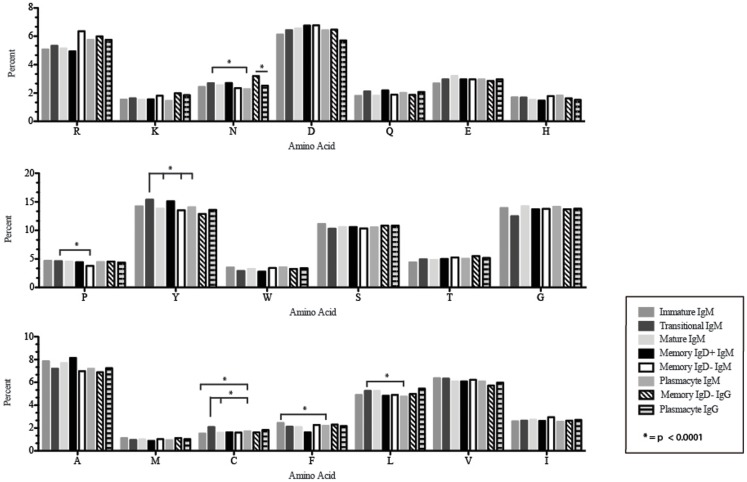
**Amino acid usage in the CDR-H3 loop of Igμ and Igγ transcripts from selected B cell populations in the blood of a normal, healthy human**. The distribution of individual amino acids is displayed. All comparisons were made using χ^2^-test or Fisher’s exact test as appropriate. Significant differences among each fraction in the different mice are indicated by asterisk: *p < 0.0001.

**Figure 9:**
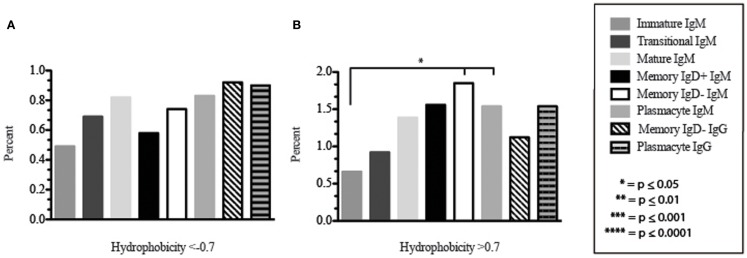
**The prevalence of highly charged and highly hydrophobic CDR-H3 loops of Igμ and Igγ transcripts from selected B cell populations in the blood of a normal, healthy human**. **(A)** Prevalence of CDR-H3 loops with an average hydrophobicity of ≤0.7 is displayed. **(B)** Prevalence of CDR-H3 loops with an average hydrophobicity of >0.7 is displayed. The normalized Kyte–Doolittle hydrophobicity scale (48) and normalized by Eisenberg (49) has been used to calculate average hydrophobicity (23). Prevalence is reported as the percent of the sequenced population of unique, in-frame, open transcripts from each B lineage fraction. All comparisons were made using χ^2^-test or Fisher’s exact test as appropriate. Significant differences among each fraction in the different mice are indicated by asterisks: *p ≤ 0.05, **p ≤ 0.01, ***p ≤ 0.001, ****p ≤ 0.0001.

Amino acid usage in the CDR-H3 loops expressed by these immature B cells varied within a narrow range. When compared to transitional cells, immature B cells used less arginine, asparagine (*p* = 0.02), aspartic acid (0.04), glutamine (*p* = 0.009), glutamic acid (*p* = 0.02), tyrosine (*p* = 0.002), threonine (*p* = 0.0039), cysteine (*p* < 0.0001), and leucine (*p* = 0.02) (Figure 8). As a result of the decrease in the use of hydrophobic and hydrophilic amino acids, the immature repertoire exhibited the lowest prevalence of highly hydrophobic (hydrophobicity >0.7) CDR-H3 loops (*p* < 0.05) and the lowest prevalence of the highly hydrophilic (hydrophobicity ≤0.7) CDR-H3 loops of the six subsets examined (Figure 9).

### The transitional B cell repertoire is characterized by the longest CDR-H3 loop length, increased use of D2–2, and increased use of tyrosine

Of the six subsets examined, the transitional CDR-H3 repertoire was the most heavily enriched for longest CDR-H3 loops (Figure 2). This bias for increased length reflects greater preservation of V(D)J gene segment sequence (Figure 2). Conversely, transitional B cell CDR-H3s were enriched for N nucleotide addition, averaging total 19.68 nucleotides and 9.75 nucleotides at the D → J junction (Figure 2). This was the first in a general pattern of diminishing N addition with maturation. Compared to the immature B cell fraction, there was a significant decrease for V_H_1 family gene segments (*p* < 0.001) (Figure 3). By V gene segment, the use of V1–69, V2–26, V3–7, V3–11, V3–15, V3–21, V3–30, V3–33, V3–NL1, V4–28, V4–31, V4–61 was greater than in immature B cells, whereas use of V1–2, V1–3, V1–8, V1–18, V1–24, V1–46, V1–58, V2–5, V3–9, V3–21, V3–23, V3–30–3, V3–48, V3–66, V3–73, V3–74, V4–34, and V4–39 was decreased (Figure 4). The transitional B cell CDR-H3 loop utilized higher levels of D_H_6 gene segments (not significant), with lower levels of D_H_5 (*p* = 0.005) than immature B cells (Figure 5). By D gene assignment, a significant increase in D1–1 (*p* = 0.09) and D2–2 (*p* = 0.0002) usage in transitional B cells was observed when compared with the immature fraction, with a compensatory decrease in D1–26 (*p* = 0.01), D2–15 (*p* < 0.0001), D3–10 (*p* = 0.005), D4–23 (*p* = 0.0026), and D5–12 (*p* = 0.0004) (Figure 6). The use of J_H_6 (*p* = 0.0008) was greater than in immature B cells, while the use of J_H_4 (*p* = 0.01) and J_H_5 (*p* = 0.09) was decreased (Figure 7).

CDR-H3 loops of these transitional cells used more arginine, lysine, asparagine (*p* = 0.02), aspartic acid (*p* = 0.04), glutamine (*p* = 0.009), glutamic acid (*p* = 0.02), tyrosine (*p* = 0.001), threonine (*p* = 0.003), cysteine (*p* < 0.0001), and leucine (*p* = 0.02), while using less tryptophan, serine, glycine, alanine, methionine, and phenylalanine than immature B cells (Figure 8). Of the six subsets studied, transitional B cells exhibited the higher prevalence of charged sequences as compared to the immature fraction (Figure 9). The contrast to the immature population was the most striking, suggesting specific gain of charged CDR-H3s in the transition from the immature to the transitional B cell stage. Conversely, the prevalence of highly hydrophobic CDR-H3s increased when compared to the immature B cell fraction.

### The mature B cell subset demonstrates a decrease in the usage of DH2 and JH6, and an increase in the percentage of highly hydrophobic and charged CDR-H3 loops

The mature B cell population was at the median for total CDR-H3 length and for the relative contributions of germline (Figure 2). Conversely, mature B cell CDR-H3s were enriched for N nucleotide addition, averaging 18.22 nucleotides total and 10.22 nucleotides at the V → D junction (Figure 2). In comparison to the transitional B cell repertoire, mature B cells exhibited similar expression of V_H_ family gene usage (Figure 3). An increase in V4–59 (*p* = 0.01) and a decrease in the use of V4–61 (*p* < 0.0001), respectively, were observed when compared to the transitional and mature fractions (Figure 4). Use of the D_H_2 (*p* = 0.01) family in general, and the D2–2 gene segment (*p* = 0.01) in particular, was lower than in transitional cells (Figures 5 and 6). There was an increase in the use of J_H_1 (*p* = 0.0004) with a decrease in the use of J_H_6 (*p* = 0.002) (Figure 7).

CDR-H3 loops demonstrated an increase in the use of glutamine (*p* = 0.007), with a decrease in tyrosine (*p* = < 0.0001), cysteine (*p* = 0.0001), and valine (*p* = 0.04) (Figure 8). As a result, the mature B cell repertoire was enriched for the use of hydrophobic and charged CDR-H3 loops when compared with immature and transitional subsets (Figure 9).

### Memory IgD^+^ and IgD^−^ B cells display divergent Igμ repertoires

The Igμ repertoires of the memory IgD^+^ and memory IgD^−^ blood B cells were distinguishable and divergent from both mature B cells and from each other. The memory IgD^+^B cell CDR-H3 region exhibited a greater contribution of germline D_H_ and J_H_ gene sequences than memory IgD^−^ (Figure 2). Memory IgD^+^ B cells used V_H_4 (*p* = 0.008) family gene segments more frequently than memory IgD^−^ B cells, and V_H_1 (*p* = 0.03) family gene segments less frequently. The memory IgD^−^ B cells used V_H_1 (*p* = 0.03) gene segments more frequently and V_H_4 (*p* = 0.03) gene segments less frequently than mature B cells (Figure 3). By individual gene V_H_ gene segment, the most prominent differences between memory IgD^+^and IgD^−^ reflected increased use of V3–23 (*p* = 0.0003), V4–34 (*p* = 0.001), V4–61 (*p* = 0.004) in the former, and decreased use of V1–8 (*p* = 0.002) and V4–74 (*p* = 0.02) in the latter (*p* < 0.0001) (Figure 4), with the exception of V4–31 (*p* = 0.02, memory IgD^−^) and V4–59 (*p* = 0.02, memory IgD^+^ and *p* = 0.01, memory IgD^−^), which was increased among mature B cells (Figure 4).

Igμ from memory IgD^+^ B cells used D3 (*p* = 0.01) family D_H_ gene segments less frequently than memory IgD^−^ cells (Figure 5). When compared with mature B cells, the memory IgD^+^ Igμ repertoire also used D2 and family D_H_ gene segments more frequently and D3 family D_H_ gene segments less frequently (not significant). Finally, memory IgD^−^ B cells appeared to use D3 family D_H_ gene segments more frequently than mature B cells, although this preference did not achieve statistical significance. By individual D_H_ gene segment, the memory IgD^+^ Igμ repertoire displayed increased use of D6–13 (*p* = 0.01); and a decrease in use of D3–3 (*p* = 0.01) (Figure 6). Divergent usage of J_H_2 and J_H_6 was also observed (Figure 7). The memory IgD^+^ Igμ repertoire used J_H_6 more frequently than the memory IgD^−^ (*p* = 0.001) or mature B cell Igμ repertoire (*p* = 0.009); and J_H_2 (*p* < 0.0001) less frequently than memory IgD^−^. J_H_ usage in the memory IgD^−^ Igμ repertoire was very similar to that observed in mature B cells, with the exception of an increase in memory IgD^−^ J_H_2 usage as compared to the mature B cells (*p* = 0.007) (Figure 7).

**Figure 10:**
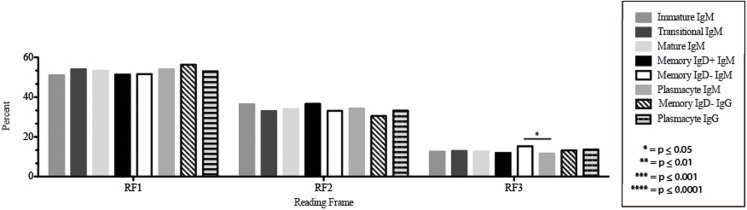
**D_H_ reading frame usage in Igμ and Igγ transcripts from selected B cell populations in the blood of a normal, healthy human**. The percent of sequences using members of the specified D_H_ family members in reading frames 1, 2, or 3 among in-frame sequences cloned from the peripheral blood from immature B cells through plasmacytes are displayed. All comparisons were made using χ^2^-test or Fisher’s exact test as appropriate. Significant differences among each fraction in the different mice are indicated by asterisks: *p ≤ 0.05, **p ≤ 0.01, ***p ≤ 0.001, ****p ≤ 0.0001.

The CDR-H3 loop of the memory IgD^+^ B Igμ repertoire contained more proline (*p* = 0.01), tyrosine (*p* = 0.01), and alanine (*p* = 0.005); but less arginine (*p* = 0.001), and tryptophan (*p* = 0.04) than memory IgD^−^ B cells (Figure 8). The increase in tyrosine reflected increased use of J_H_6, rather than increased use of reading frame 1. Indeed, use of reading frame 1, 2, and 3 were similar between the memory fractions (Figure 10). When compared to mature B cells, the memory IgD^+^ Igμ repertoire was similarly enriched for glutamine (*p* = 0.02) and tyrosine (*p* = 0.03), and depleted of phenylalanine (*p* = 0.01). The memory IgD^−^ Igμ repertoire also contained more arginine (*p* = 0.005) and less proline (*p* = 0.01) than the mature B cell Igμ repertoire. The memory IgD^−^ Igμ repertoire relatively contained a higher percentage of highly charged CDR-H3s (hydrophobicity >0.7) (1.85%) when compared to the Igμ repertoires of subsequent B cell fractions (Figure 9).

### The plasmacyte Igμ repertoire diverged from both the memory IgD^+^ and IgD^−^ Igμ repertoire, as well as from the mature B cell Igμ repertoire

In comparison to the other Igμ and Igγ repertoires, the CDR-H3 component of the plasmacyte Igμ repertoire exhibited the fewest N nucleotides at both the V → D and D → J junctions, respectively. As a result, not only the Igμ repertoire relatively enriched for germline V(D)J sequence, but also exhibited the shortest average length (Figure 2).

By V_H_ family, plasmacytes exhibited higher usage of V_H_4 than either memory B cell population, and lower usage of V_H_2, V_H_3, and V_H_5 (Figure 3). These differences were most affected by increased use of V4–34 (*p* = 0.007, *p* < 0.0001) when compared to both the memory IgD^−^ and IgD^+^Igμ repertoires and decreased use of V5–51 (*p* = 0.01) when compared to the memory IgD^−^ Igμ repertoire (Figure 4).

The distribution of D_H_ gene family usage among the plasmacyte Igμ repertoire was similar to that of the mature B cell Igμ repertoire, but differed for individual families with the two memory B cell Igμ repertoires. There were no statistically significant differences in the use of D_H_ gene segments between the memory IgD^+^ and the plasmacyte Igμ repertoires. When compared to the memory IgD^−^ Igμ repertoire, the plasmacyte Igμ repertoire used D_H_5 gene segments more frequently (*p* = 0.04) (Figure 5). By individual D_H_ gene segment, plasmacytes used D6–25 more frequently (*p* = 0.006) and D7–27 less frequently (*p* = 0.03) than mature B cells. Plasmacytes used D6–25 more frequently (*p* < 0.0001), and D4–11 (*p* = 0.01), D6–13 (*p* = 0.04), and D6–6 (*p* = 0.03) less frequently than the IgD^+^ memory Igμ repertoire. Finally, plasmacytes used D5–24 (*p* = 0.007) and D6–25 (*p* = 0.0001) more frequently, and D3–9 (*p* = 0.03) less frequently than the memory IgD^−^ Igμ repertoire (Figure 6).

By J_H_ gene segment, the plasmacyte Igμ repertoire displayed similar levels of J gene segments when compared to the mature B cell Igμ repertoire. Plasmacytes expressed higher levels of J_H_2 (*p* = 0.01), J_H_4 (*p* = 0.01); and lower levels of J_H_6 than memory IgD^+^ B cells (*p* = 0.0004). Finally, plasmacytes expressed lower levels of J_H_2 (*p* = 0.04) than memory IgD^−^ B cells (Figure 7).

When compared with the mature B cell Igμ repertoire, plasmacytes expressed lower levels of asparagine (*p* = 0.02), alanine (*p* = 0.01), and leucine (*p* = 0.007) in the CDR-H3 loop. When compared with memory IgD^+^ B cells, plasmacytes expressed lower levels of asparagine (*p* = 0.001), aspartic acid (*p* = 0.02), glutamine (*p* = 0.04), tyrosine (*p* = 0.001), and alanine (*p* = 0.0002); and higher levels of tryptophan (*p* = 0.02) and phenylalanine (*p* = 0.01). When compared with the memory IgD^−^ Igμ repertoire, plasmacytes expressed lower levels of arginine (*p* = 0.02), lysine (*p* = 0.009), and isoleucine (*p* = 0.02) (Figure 8).

**Figure 11:**
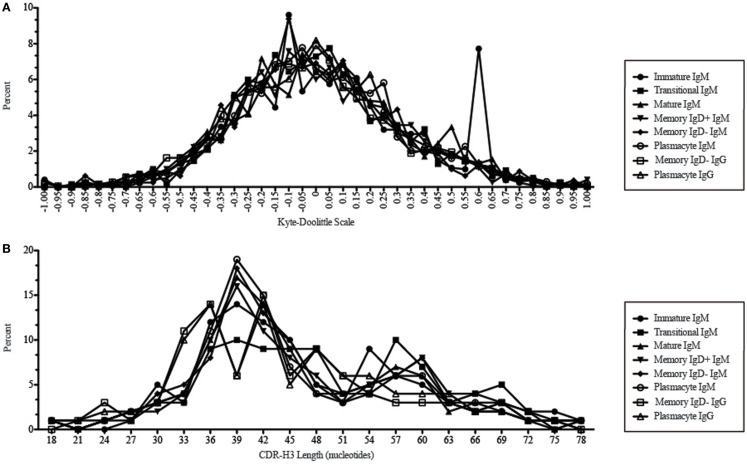
**CDR-H3 loop charge and length as a function of B cell development in the peripheral blood of this adult subject**. **(A)** Distribution of CDR-H3 hydrophobicities in Igμ and Igγ transcripts from peripheral blood as a function of B cell development. The Kyte–Doolittle hydrophobicity scale (48) as normalized by Esienberg (49) has been used to calculate average hydrophobicity (30). Although this scale ranges from −1.3 to +1.7, only the range from –1.0 (charged) to +1.0 (hydrophobic) is shown. Prevalence is reported as the percent of the sequenced population of unique, in-frame, open transcripts from each B lineage fraction. **(B)** Distribution of CDR-H3 lengths in nucleotides of μ and γ H chain transcripts is displayed.

When comparing the relative prevalence of either highly charged or highly hydrophobic CDR-H3 loops, plasmacytes were enriched for charged CDR-H3 loops (0.84%) in comparison to the five other Igμ repertoires (Figures 9 and 11). The distribution of highly hydrophobic CDR-H3 loops decreased in plasmacytes (1.54%) as compared to memory IgD^−^ B cells (1.85%), and returned to the comparable levels of memory IgD^+^ B cells (1.56%) (Figure 9).

### The plasmacyte Igγ repertoire diverged from IgD^−^ IgD^+^ memory B cells

The *Igγ* repertoires expressed by memory IgD^−^ B cells and plasmacytes were distinguishable and uniquely different from each other. While the average length and V(D)J gene segment length was very similar between the memory IgD^−^ and plasmacytes, differences in the N-region additions were observed. The memory IgD^−^ B cell CDR-H3 region exhibited a greater number of N nucleotide addition at the V-D junction (10.56 nucleotides) as compared to the plasmacytes. Conversely, plasmacytes contained more N nucleotide addition at the D–J junction than memory IgD^−^ B cells (10.08 nucleotides) (Figure 2). Memory IgD^−^ B cells used V_H_1 (*p* = 0.03), V_H_2 (*p* = 0.0001), and V_H_3 (*p* = 0.0003) family gene segments more frequently than plasmacyte; and V_H_4 (*p* < 0.0001) family gene segments less frequently (Figure 3). This pattern is due to an increase in individual gene V_H_ gene segment, the most prominent differences between memory IgD^−^ and plasmacytes reflected increased use of V1–2 (*p* = 0.03), V1–8 (*p* = 0.003), V2–5 (*p* = 0.0003), V3–7 (*p* = 0.01), V3–15 (*p* = 0.001), V3–30 (*p* = 0.005), and V4–40–2 (*p* = 0.01), in the former, and decreased use of V4–4 (*p* = 0.0007) and V4–34 (*p* < 0.0001) in the latter (Figure 4).

The memory IgD^−^ Igγ repertoire used D6 (*p* = 0.01) family D_H_ gene segments less frequently than plasmacyte Igγ (Figure 5). By individual D_H_ gene segment, the memory IgD^−^ Igγ repertoire displayed increased use of D5–24 (*p* = 0.005) and decreased use of D2–21 (*p* = 0.03) (Figure 6). The memory IgD^−^ Igγ repertoire used J_H_6 less frequently than plasmacytes (*p* = 0.0006) (Figure 7).

The CDR-H3 loop of the memory IgD^−^ Igγ repertoire contained more asparagine (*p* < 0.0001) and aspartic acid (*p* = 0.01); but less tyrosine (*p* = 0.04), cysteine (*p* = 0.03), and leucine (*p* = 0.01) than plasmacyte Igγ (Figure 8). The plasmacyte Igγ repertoire was relatively enriched for hydrophobic amino acids, which was reflected by a higher percentage of hydrophobic CDR-H3s (hydrophobicity > 0.7) (1.54%) when compared to the memory IgD^−^ (1.12%) (Figure 9).

The Igμ and Igγ repertoires of analyzed cell types expressed similar distribution of D_H_ reading frames, with reading frame 1 having greatest preference, followed by reading frame 2 and reading frame 3 (Figure 10), while the μH chain plasmacytes used reading frame 3 less likely than memory IgD^−^ B cells (*p* = 0.03) (Figure 10).

## Discussion

In both mice and humans, the composition of the antibody repertoire varies by ontogeny and by developmental stage (29, 37, 50). In order to study this process in detail, we developed a series of tools to evaluate the development of the repertoire in mice. This approach enabled us to identify constraints on V(D)J gene segment preference and CDR-H3 composition that are first established in early B cell progenitors, and then focused as the B lineage cells pass through various developmental checkpoints. The constraints are a reflection of the specific sequence from the contributing gene segments that vary in usage as a function of development (29, 30, 51–55).

Differences in the individual V–D–J gene usage, length, and amino acid composition of the adult human germline repertoires from peripheral blood and specific tissues have been previously reported (37, 50, 56–62), but comparative studies of repertoire development in human blood have been sparse. The difficulty of study is compounded by the enhanced variability of the human repertoire when compared to mice, especially in CDR-H3. This reflects both a greater diversity of the germline sequence of the D_H_ gene segment sequences and an increase in the extent of N addition when compared to mouse. In this work, we sought to use the same tools we had developed for the study of the mouse repertoire to perform a comparative analysis of the expressed in both the Igμ and Igγ repertoires in the blood of a normal, healthy human female in order to gain insight into the forces that shape the repertoire during its passage through the different stages of B cell ontogeny.

While similarities have been reported between the frequency of naïve and memory B cell repertoire usage of the V–D–J gene segments (58, 61, 62), our analysis focuses on a more detailed examination of the repertoires. Our results of low J_H_1 and J_H_2 usage across B cell development is consistent with previous published reports of low J_H_1 and J_H_2 usage in transitional, naïve, switched, and IgM memory B cell repertoires (Figure 7) (61). Altered expression of individual V_H_ gene segments have been previously also reported in the transitional, naïve, switched, and IgM memory B cell antibody repertoires (61). As in mice, we found changes in V(D)J gene segment usage and CDR-H3 hydrophobicity in the progression from immature to transitional to mature (Figures 3, 5, 7, 9, and 11). These observations support the view that the B cell receptor repertoire continues to be selected throughout early and late B cell development in the peripheral blood. Unlike mice, however, the prevalence of highly charged CDR-H3 loops increased during maturation from the immature to mature cell subsets and memory IgD^−^ to plasmacyte subsets (Figure 9). Also unlike mice, the prevalence of highly hydrophobic CDR-H3 loops also increased in our human study subject. This may reflect a greater tolerance or preference for the use of amino acids encoded by hydrophobic D_H_ reading frame 2 in human B cells exposed to self and non-self antigens (35%) when compared to mice (10%), or a property specific to this particular individual, since patterns of regulation have been shown to differ in mouse strains (Figure 10) (34, 63).

We observed a decrease in the length of CDR-H3 during maturation (Figures 2 and 11). This appears to be part of a continuum of focusing CDR-H3 length in developing B cells in the bone marrow (50) and has been observed by others, as well (61). The use of long CDR-H3 loops has been previously associated with enhanced autoreactivity and polyreactivity (38, 64–66), which are presumably the features of this component of the antibody repertoire that somatic selection are designed to minimize by apoptosis or anergy.

Selection past the mature B cell stage is considered to reflect both endogenous and exogenous antigen exposure. In this regard, the most striking findings of our study were the distinctly different repertoires expressed by the memory IgD^+^Igμ, the memory IgD^−^ Igμ, and Igγ repertoires; and the plasmacyte Igμ and Igγ repertoires. We did not sort memory B cells or plasmacytes by Igμ or Igγ expression, but were able to identify unique Igμ or Igγ reads through the use of Igμ and Igγ specific primers.

The memory IgD^+^ and memory IgD^−^ Igμ repertoires displayed differences in virtually all of the features of the repertoire that we evaluated, including V(D)J usage, N addition, D_H_ reading frame usage, CDR-H3 length, CDR-H3 loop amino acid content, and CDR-H3 hydrophobicity (Figures 3–11). Differences in IgD^+^ and IgD^−^ Igμ repertoires in V_H_1 gene family usage (*p* = 0.03) (Figure 3) have been reported previously (61). We observed a similar decrease in usage of V_H_3–23 (*p* = 0.0003) between the memory IgD^+^ and memory IgD^−^ Igμ repertoires (Figure 4) (61). Differences between these two memory Igμ repertoires were further enhanced by altered amino acid usage, especially an increase in arginine (*p* = 0.001) and decrease of tyrosine (*p* = 0.01) in the memory IgD^−^ Igμ cell subset as compared to the memory IgD^+^ Igμ cell subset (Figure 8) (61). The memory IgD^−^ Igμ repertoire exhibited enhanced use of charged amino acids and hydrophobic amino acids (Figure 8). As a result, there was a higher percentage of CDR-H3s with excess charge when compared to the memory IgD^+^ Igμ repertoire (Figure 9). These observations are consistent with a previous report showing that IgD^+^ memory cells had levels of negatively charged amino acids comparable to transitional and naïve B cells, while switched memory had more negatively charged residues (Figures 8 and 9) (61).

The vast majority of the IgD^+^ memory B cell pool also expresses IgM, whereas the IgD^−^ pool expresses class-switched Ig in addition to IgM. Memory B cells expressing both IgM and IgD are considered to be the circulating equivalents of the marginal zone B cell subset in mice; whereas memory B cells restricted to IgM production are considered to represent the more conventional B cell pool, which also is the primary source for class-switched B cells. Thus, our observations regarding the differences in repertoire between the IgD^+^ and IgD^−^ memory B cell pools fit well within the view that the IgM^+^IgD^+^ and IgM^+^IgD^−^ memory subsets are the products of very different immune responses. In this regard, the marginal zone-like repertoire expressed by our female study subject diverges from the marginal zone repertoire expressed in BALB/c mice in that BALB/c appears tolerant for charged CDR-H3s (35), whereas in our study subject B cells expressing charged CDR-H3s were more likely to be found in the memory IgD^−^ population. Whether this difference represents a common difference between human and mouse, or reflects variation within the outbred human population is unclear and will require analysis of additional study subjects.

The plasmacyte pool represents the products of recently activated mature B cells as well as memory IgD^+^ and IgD^−^ B cells that have been reactivated. This observation may explain why the plasmacyte repertoire appears intermediate between the memory IgD^+^ and IgD^−^ repertoires and the mature B cell population. At present, the tools do not exist to separate plasmacytes by derivation. Moreover, the content of the memory and plasmacyte populations are likely to have been heavily influenced by several decades exposure to a variety of endogenous and exogenous antigens as well as by the anatomic niches in which the disparate subsets reside. Our study focused on bulk sequencing rather than analysis of repertoire in cells that were isolated by specific antigen reactivity, thus we cannot define the precise nature of the response to specific antigens. However, the most striking difference between the plasmacyte population and the other subsets in bulk was the decrease in the contribution of N nucleotides to the final product. Coupled with the observation that the greatest contribution of non-germline encoded nucleotides among the six subsets studied was found in the immature B cell fraction, final enrichment for germline V(D)J sequence among plasmacytes supports the view that the germline V domain repertoire has been selected by evolution for maximal advantage in responding to antigen (34–36).

As in mouse, the repertoires expressed by distinct B cell subset appear to differ in human. Sequencing of unsorted B cells from the blood is thus likely to yield an incomplete view of what is actually happening in the immune response of the individual. Our findings support the view that determination of whether diseases of immune function reflect abnormal regulation of these various B cell subsets will require considerable effort to perform deep sequencing of sorted cells from a variety of healthy individuals and patients with immune-mediated disorders (14, 38).

## Conflict of Interest Statement

The authors declare that the research was conducted in the absence of any commercial or financial relationships that could be construed as a potential conflict of interest.
